# “*Then and Now*,” Mapping the 25 Year Evolution and Impact of North American Vascular Biology Organization Science Through Publications of its Founding and Current Members

**DOI:** 10.3389/frma.2020.591090

**Published:** 2020-11-12

**Authors:** Zorina S. Galis, Bruce W. Herr, Santoshmurti S. Daptardar, Medina Sydykanova, Katy Börner

**Affiliations:** ^1^ National Heart Lung and Blood Institute (NHLBI), Bethesda, MD, United States; ^2^ Cyberinfrastructure for Network Science Center, Luddy School of Informatics, Computing, and Engineering, Indiana University, Bloomington, IN, United States

**Keywords:** scientometrics, bibliometrics, science of science, science maps, network science, research assessment, impact assessment

## Abstract

Scholarly organizations bring together experts to move forward specific areas of scientific endeavor. More than 5,000 scholarly societies exist world-wide yet little is known about the composition, evolution, and collaboration of experts within these organizations. This study presents general methods to study the evolution of a biomedical organization and its impact over time. Methods are illustrated in a case study that aims to capture the “Then and Now” science of the North American Vascular Biology Organization (NAVBO). Publications authored by the founding members who came together to create NAVBO in 1994 are compared with publications by those representing the society 25 years later. Google Scholar data was compiled for NAVBO members registered in 1994 (n = 70) and in 2019 (n = 465), some members being present in both data sets (n = 22). The 501 unique NAVBO members had more than 76,000 papers cited over 4,400,000 times. Several characteristics associated with the NAVBO members’ output were revealed, including their high productivity, the high impact of their publications, and the predominant contribution of relatively small laboratories, as suggested by a low average number of authors per publication. To understand how NAVBO fostered scientific collaborations and exchanges of expertize, data was analyzed and visualized to show the evolution of member co-author networks. The UCSD Map of Science was used to communicate the evolution of scientific topics covered by NAVBO members helping to create a global picture of NAVBO’s science “then and now.” All workflows are available online in case other scholarly organizations would want to use them to map their own evolution and impact; and meta studies that communicate the inner workings of these important scholarly efforts can be conducted.

## Introduction

The structure and evolution of science is commonly studied using publication, funding, and author data for a specific topic area, institution, geo-region, or time frame (Janssen et al., 2006; Börner, 2010; Bruer, 2010; Börner, 2015). Hundreds of examples and more than 1,000 references to prior work on science of science studies as well as detailed explanations of temporal, geospatial, topical, and network analysis and visualization workflows can be found in (Börner, 2010; Börner, 2015).

This study is unique in that it studies the evolution and impact of a 25-year-old scholarly society, the North American Vascular Biology Organization (NAVBO). NAVBO is a member-driven organization for vascular biologists and other scientists from different backgrounds that was created in 1994 to provide a forum for sharing knowledge related to the field of vascular biology research through facilitating in-person and online interactions, conducting various training and scientific programs, organizing annual and specialty meetings and workshops. The society provides many different opportunities for healthy discussions, collaborations, mutual education, and communication within the vascular biology community, that all together lead ultimately to increased impact of research and discovery within this field.

In 2019, NAVBO had its 25th anniversary and society members aimed to capture the “Then and Now” of NAVBO science through the lens of publications of the founding members who created NAVBO, and those representing today’s organization as it celebrated its Silver Jubilee.

Prior studies used Web of Science publication data to showcase the evolution of scholarly networks and different lines of research (Janssen et al., 2006) or to study the impact of funding on the progression of research fields (Bruer, 2010). Here, we use Google Scholar data to capture the evolution and impact of NAVBO. Specifically, we tried to understand how NAVBO fostered collaborations by extracting and visualizing NAVBO member co-author networks and highlight the evolution of scientific topics using science maps that show what topics emerged, merged, split, and diminished during NAVBO’s initial 25 years of existence.

## Prior Work

Publication, patent, grant funding, and other scholarly data can be used to study and communicate the evolution of scientific disciplines, the impact of different funding mechanisms, or the emergence and decline of scholarly networks, among others (Börner, 2010; Börner, 2015).

For example, prior work by Janssen et al. (2006) analyzed 2,286 publications published between 1967 and 2005 on human dimensions of global environmental change such as resilience, adaptation and vulnerability; they showed that there is an increasing number of co-authoring and cross citations among papers in these three domains which indicates an integration of these different knowledge domains. Bruer (2010) was able to show the development of the cognitive neuroscience of attention using papers published in 1980–2005 and performing an in-depth study of cognitive psychology, single cell neurophysiology, neuropsychology, and evoked potential research resulting in visualizations that showcase the evolution of these research areas. Stipelman et al. (2014) mapped the impact of transdisciplinary research via a visual comparison of investigator initiated R01 funded research and topically and temporally comparable set of team-based, research center funded tobacco use research publications; study results revealed that publication results from the transdisciplinary research centers spread across the topic map of science more rapidly and more comprehensively than R01 funded research results.

## Data

NAVBO had 70 members in 1994, the year when it was created, whereas by 2019, the organization had grown to 465 members. We identified 22 members who have been with NAVBO for all 25 of its years, thus the total list of unique members is 501. Compiling a dataset that covers all major works by more than 500 authors is non-trivial. We initially tried using Web of Science (https://webofknowledge.com) and Scopus data (https://scopus.com) but did not achieve the coverage (many NAVBO members missed major works) nor the level of author-disambiguation (e.g., for members with common names or author name changes due to marriage) desired for this study. To compile a high coverage, correctly disambiguated dataset, a call to the entire current NAVBO membership was made to update their Google Scholar profiles for this study. For each of the 501 NAVBO members, all publications were downloaded based on their Google Scholar profiles using the Publish or Perish software by Harzing (2007). If members did not have a profile, publications were extracted using Google Scholar Query in Publish or Perish by entering the member’s name and affiliation as a parameter. For all but 12 NAVBO members, publication data was available via Google Scholar. The use of Google Scholar data plus human review and curation used here will make it easier for other teams to reproduce the studies as anyone can freely use this site while Web of Science and Scopus are for-pay services that few have access to.

Custom code was used for data processing and cleaning, all code and documentation is available via GitHub [https://github.com/cns-iu/vascular-team-map]. Processing steps are as follows: First, publications by all members are merged into a single file and the NAVBO member for each publication is listed in a newly created “Source Author” field. Second, a co-authorship network was extracted by using NAVBO co-authors present in “Source author” column that have publications with the same “Title,” “Year,” and “Source” field value; fields like “number of citations” and “year” were retained and used for color and size coding. Finally, journal name, publisher and year columns are kept for generating map of science visualizations using the Sci2 Tool (Sci2 Team, 2009).

## Methods

To understand the topical evolution of scientific topics, three UCSD Map of Science and Classification System visualizations were created using the Sci2 Tool (Sci2 Team, 2009) to cover the period from 1990 to 1994, 1990 to 2004, and 1990 to 2019. The UCSD Map of Science organizes more than 25,000 journals/conference venues into 554 sub-disciplines, which are further aggregated into 13 primary scientific disciplines that are laid out using a reference or “basemap” (Börner et al., 2012). Each discipline is labeled and color-coded in the map. To create a proportional symbol data overlay, journal names of publications are mapped to sub-disciplines using a predefined lookup table. Each colorful circle represents a unique subdiscipline and is sized by how many scientific articles are present within that subdiscipline. All the science maps use the same scaling factor of 0.003 to support comparison.

To study the evolution of co-author networks and topical coverage of publications by NAVBO members, two types of visualizations were created. First, a co-author network was extracted using the code provided on GitHub and processed using the Sci2 Tool (Sci2 Team, 2009). The undirected, weighted network was preprocessed in Sci2 using MST PathFinder Network Scaling to identify major backbones and make the networks more legible. Force Atlas two layout in Gephi (Jacomy et al., 2014) was used to visualize the networks. All co-author networks use the very same x-y positions for author nodes to ease comparison. This “basemap” was computed by generating a network layout for the complete 30-years co-author network, saving out the positions of all nodes, and using these author node positions for the layout of all co-author networks.

In the co-author networks, each node represents a NAVBO member, node size denotes number of papers, and node color represents the number of citations of the author. Each edge represents a joint collaboration (i.e., two NAVBO members co-authoring a paper); edges are thickness coded by the number of joint papers and color coded by the publication year of the first joint publication with darker colors denoting older collaborations. Only the top 20 highly cited authors are labeled in each co-author network. All co-author networks use the very same color and size coding to ease comparison.

A total of four co-author networks are featured in this paper. The first set of three figures shows the evolution of the co-author network over three periods: 1990–1994, 1990–2004, and 1990–2019. The network for 1990–1994 shows only authors that published papers during that very time span. The network for 1990–2004 features additional nodes for authors that published in 1995–2004 and the size of nodes and edges reflects the number of papers and citations for the entire time span, 1990–2004. The third network features all authors active in the full 30-years time span and the node and edges sizes and colors now represent all years, 1990–2019. A fourth network shows the co-authorship network for the last 15 years the dataset covers, 2005–2019; only authors active during those years are shown and the color and size coding of nodes and edges reflects the number of papers and citations during that 15-year period. The network was included to support comparison of the initial years of the NAVBO with the most recent 15 years of NAVBO.

## Results

**FIGURE 1:**
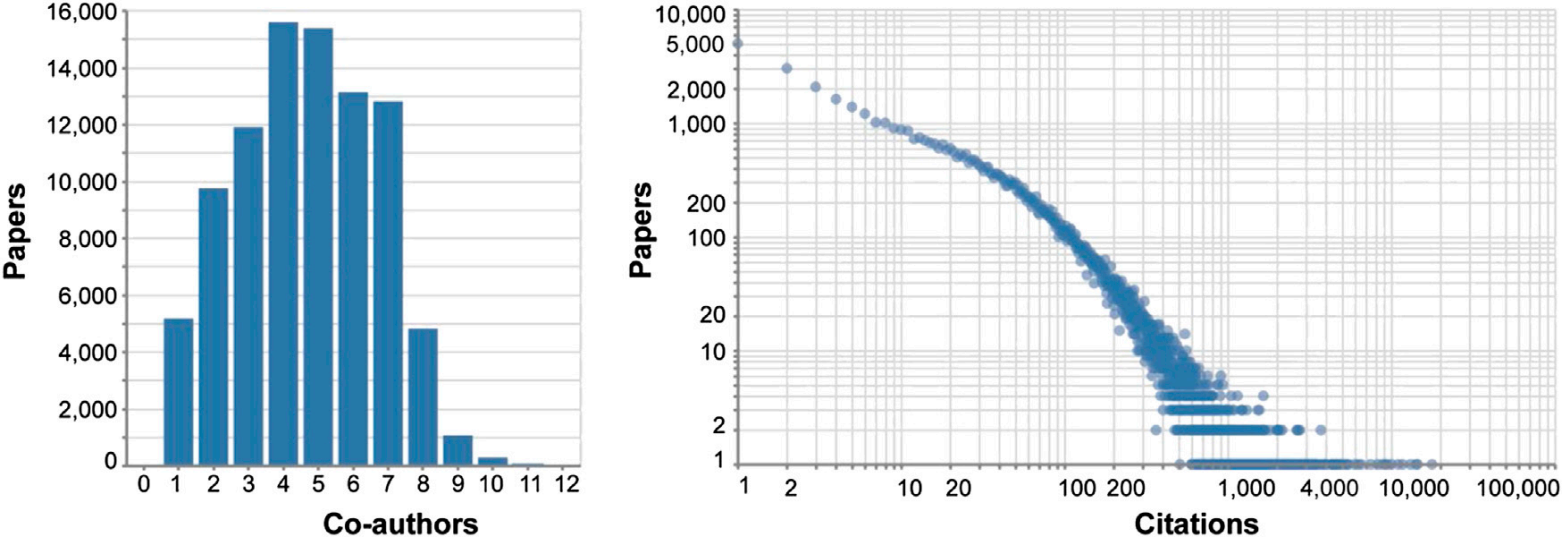
Number of NAVBO member co-authors per publication **(left)**. Distribution of number of citations per publication on log-scale **(right)**.

We first examined author productivity and scientific impact. The analysis indicated a total of over 76,000 papers written by 501 unique NAVBO members were cited over 4,400,000 times. The distribution of the number of papers based on the number of co-authors for the 1990–2019 period is given in Figure 1; most papers have one to eight co-authors and only 13 papers have more than 12 authors (not included in the graph). The distribution suggests that most papers represent the output of single independent research laboratories or smaller teams collaborating.

**FIGURE 2:**
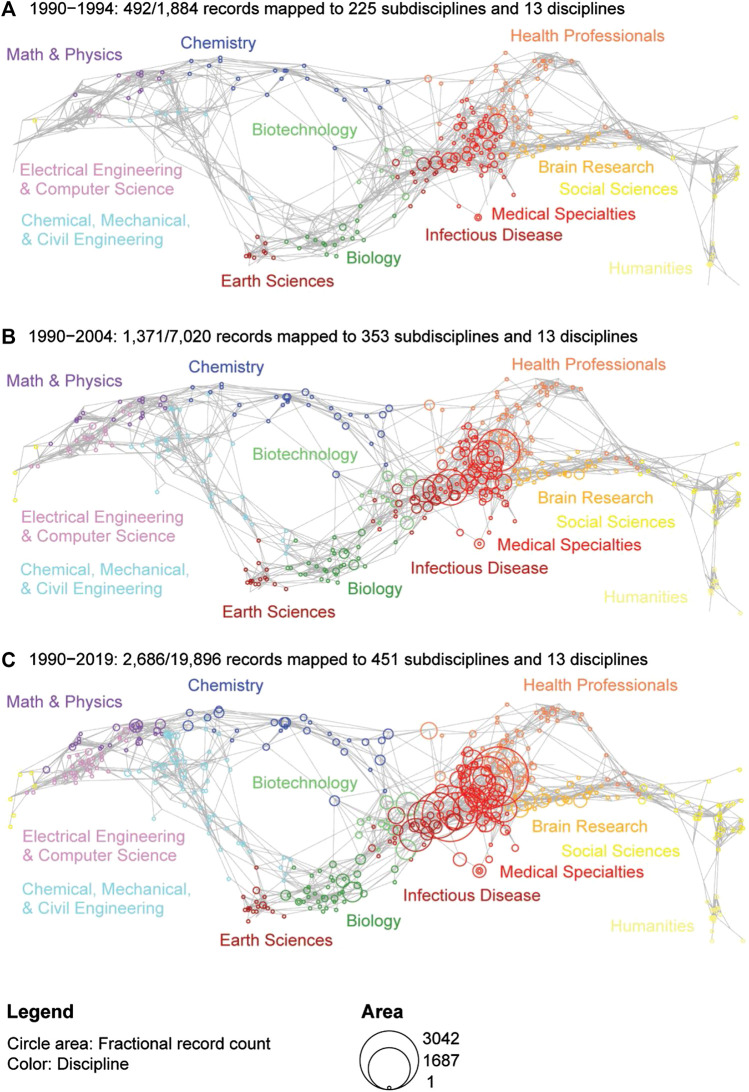
USCD Map of Science for three time periods showing the evolution of disciplines and subdisciplines associated with NAVBO publications.

The number of citations per publication in log-scale is shown in Figure 2. It documents the high impact of the NAVBO members publications: 12,596 papers have 100 or more citations and 530 papers have more than 1,000 citations. Furthermore, only 35% of all papers have no citations while the typical percentage is 50%. The top 20 authors in this cohort were cited tens of thousands of times. Only 4% of all authors that published have less than 10 publications which indicates high productivity of NAVBO members.

Next, we studied the scientific coverage and evolution of NAVBO. To investigate the areas of science most popular among the NAVBO founding members, a map of science was created using the UCSD Map of Science and Classification System for publications between 1990 and 1994, the period immediately before the creation of NAVBO, see Figures 2A. During this period, all the publications of the experts who founded NAVBO were mapped to 225 subdisciplines, suggesting that the founding members already covered a wide range of topics. Most of the publications belong to “medical specialties” followed by “infectious disease” discipline.

Figures 2B shows the map of NAVBO science during its first 15 years (1990–2004); the map covers an increased number of subdisciplines, with most topics still belonging to medical specialties followed by infectious disease. Figures 2C show the map of science for the entire time period 1990–2019; the number of subdisciplines now covers 451 out of the total of 554 subdisciplines, showcasing the highly interdisciplinary impact of the NAVBO members and their science—touching upon almost every subdisciplines of science represented in the map.

The number of NAVBO members publications grows substantially over these three time periods. The strongest growth is associated with medical specialties and infectious disease disciplines. In addition, there is a steady increase in the number of publications in scientific topics like biology, chemistry, engineering specialties, including biotechnology and computer science.

**FIGURE 3:**
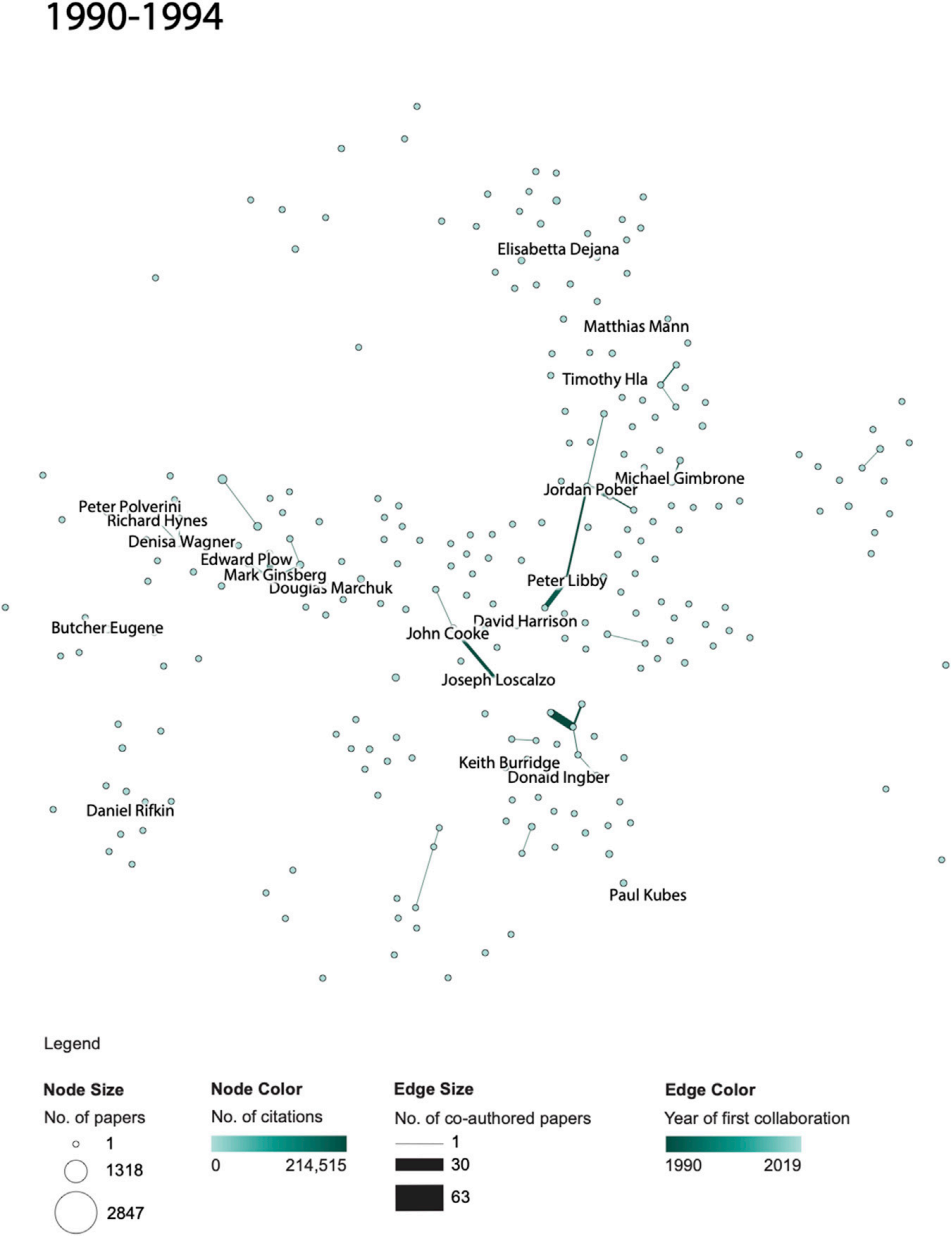
NAVBO Co-author Network during 1990–1994.

**FIGURE 4:**
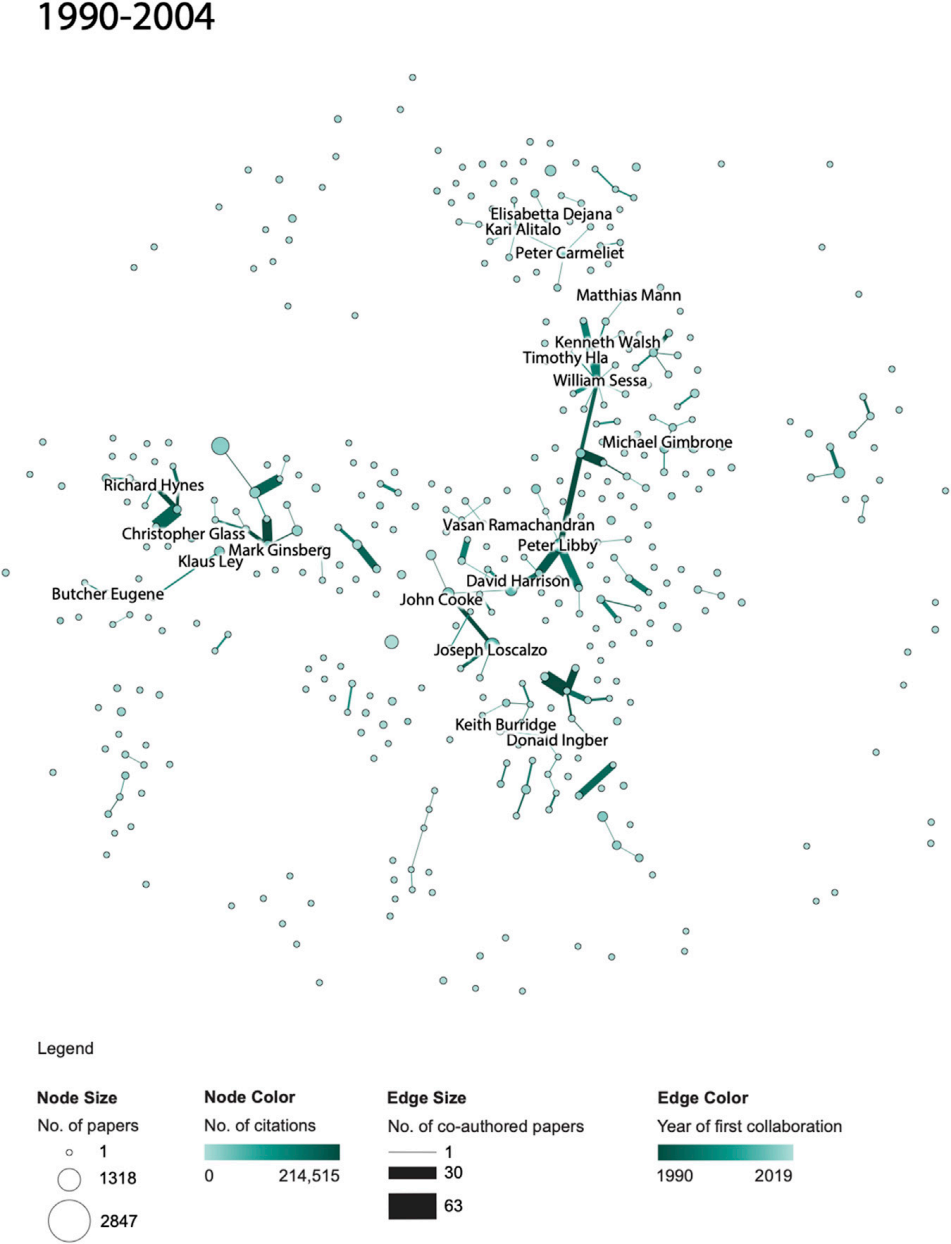
NAVBO Co-author Network during 1990–2004.

**FIGURE 5:**
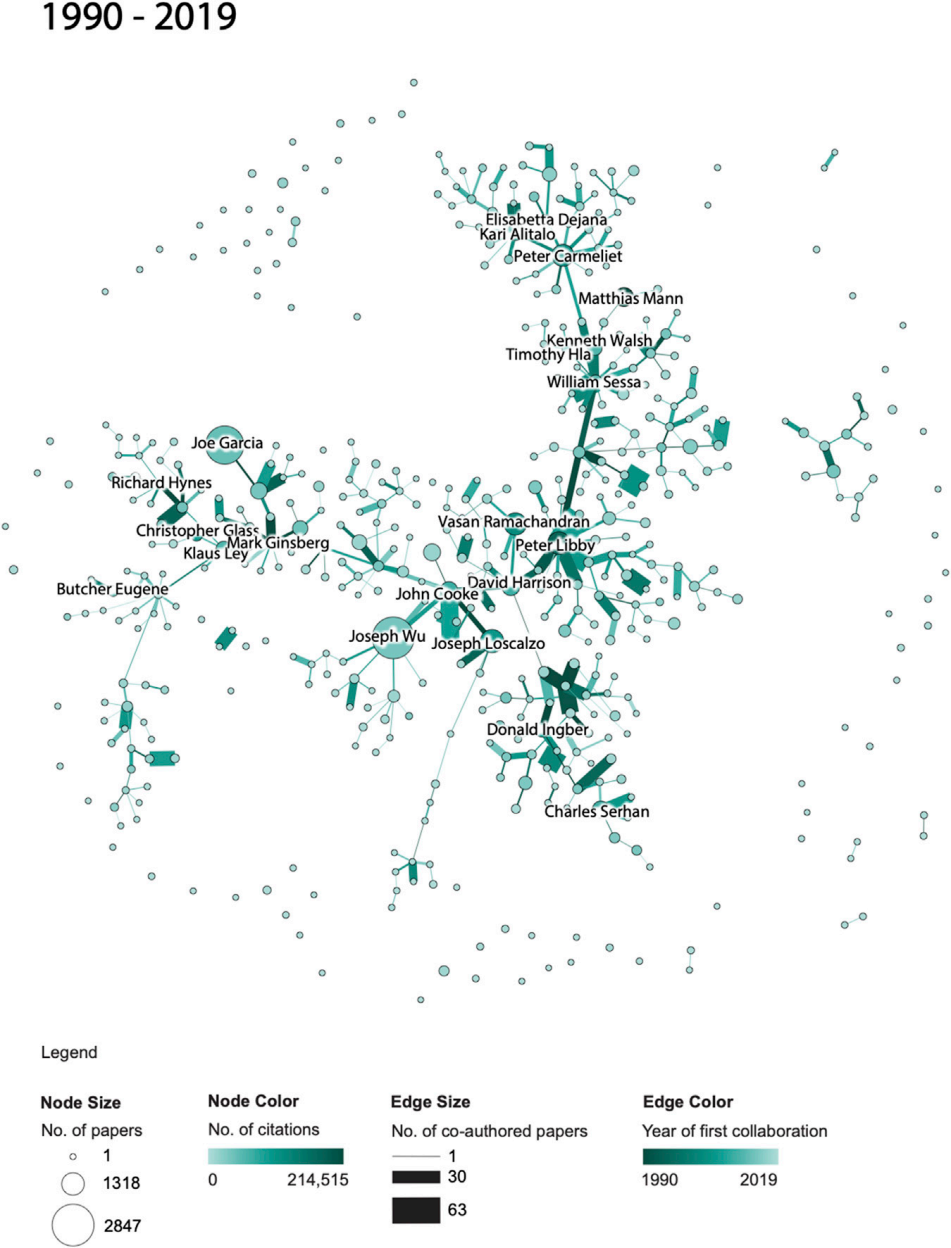
NAVBO Co-author Network during 1990–2019.

Third, we examined the evolution of author networks. The same three time periods were investigated to examine the evolution of NAVBO co-author networks over time, see Figure 3-5. The visualizations show the number of published papers and their citations for various authors, the number of co-authored papers and the year of first collaboration. A comparison of the three figures helps communicate the overall growth of authors, published papers, and collaborations among the NAVBO members during these 25 years. The analysis also highlights authors who had the highest number of collaborations and citations. Many of these members had been recognized by NAVBO with meritorious awards and have served as leaders of the organization (North American Vascular Biology Organization, 2020).

**FIGURE 6:**
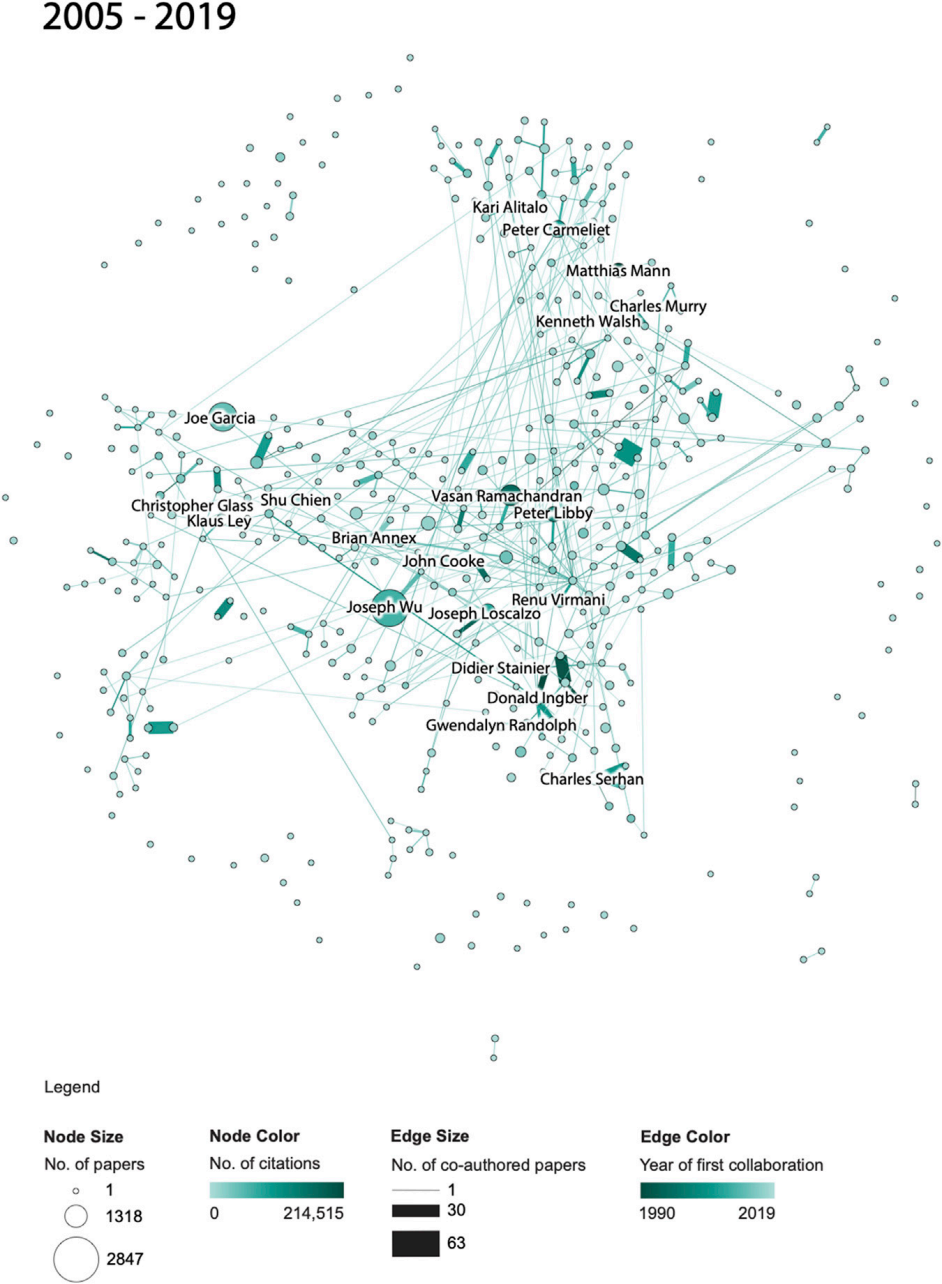
NAVBO Co-author Network for the last 15 years: 2005–2019.

While Figure 3-5 show the “cumulative” growth of the co-author network for 1990–1994, 1990–2004, and 1990–2019, it is interesting to compare the initial years of NAVBO with more recent years of NAVBO co-authoring activity. A separate analysis was run over the last 15 years of the dataset, 2005–2019 and the result can be seen in Figure 6. The figure uses the same node layout “basemap” as Figure 3-5 but only shows those authors active in the last 15 years.

When comparing the co-author network shown in Figure 6 with the initial 15 years of the dataset, see Figure 4, it becomes visible that several authors were very active in both sets; for example, *Kari Alitalo* and *Peter Carmeliet* in the top part of the network were both among the top-20 most cited authors in both time periods. *Vasan Ramachandran*, in the middle of the network, was highly cited in the earlier network but has many more publications (indicated by a larger node size) in the last 15 years. *Joseph Wu* did not make the top-20 most cited list in the earlier time span but is the author with the most papers in the recent 15 years.

The co-author relationships changed over time as well. Some relationships are strong in both time periods, some only exist in the earlier or later time period. The long edges in Figure 6 represent strong new co-authorship relations across author clusters that did not exist in the earlier years.

Note that the number of papers and citations in Figures 4, 6 sum up to the total of all papers and citations featured in Figure 5.

## Discussion

This study was performed with the engagement and participation of NAVBO members who helped update their Google Scholar profiles and interpret and substantiate the evolution of NAVBO. Although NAVBO has attracted those who self-identify as “vascular” researchers, the analysis made it obvious that their work is encompassing and has impacted a multitude of other scientific domains. In addition to revealing the high interdisciplinarity of this member-driven organization, the analysis also quantified the scientific impact and the growth of collaborations among NAVBO members.

There are a number of caveats to the analyses presented here. For instance, we made several assumptions, the main assumption being that the collective science of NAVBO members is a good representation for the science of this organization. However, it is expected that many members belong to multiple professional organizations. Plus, only founding and members in 2019 were included—not members associated with NAVBO in-between; for 12 members no publication data was available via Google Scholar. Science map generation was difficult due to the many special characters and incomplete journal names in the Google Scholar data.

Study results were shared with domain experts for examination, interpretation and evaluation. Major structure and dynamics of NAVBO were confirmed and substantiated: Many of the most prolific and impactful authors rank among those who have been recognized previously with NAVBO scientific and meritorious awards and/or had been elected in organizational leadership roles. At the same time, the data also helped understand and communicate recent developments and can guide future decisions regarding recognition of individuals or areas that may need to be highlighted or strengthened through future scientific or training programs.

## Data Availability Statement

Publicly available datasets were analyzed in this study. This data can be found here: https://github.com/cns-iu/vascular-team-map.

## Author Contributions

ZSG and KB designed the research; ZSG, SSD, and MS compiled the data; BWH, SSD, MS, and KB performed the data analyses and developed the visualizations; all authors contributed to the writing of the manuscript.

## Funding

This work is partially supported by a Humboldt Research Award and National Science Foundation Awards AISL-1713567, DGE-1735095, and DMS-1839167.

## Disclaimer

Any opinions, findings, and conclusions or recommendations expressed in this material are those of the author(s) and do not necessarily reflect the views of the NHLBI or the NSF.

## Conflict of Interest

The authors declare that the research was conducted in the absence of any commercial or financial relationships that could be construed as a potential conflict of interest.
